# Mesenchymal Stem Cells Can Both Enhance and Inhibit the Cellular Response to DNA Immunization by Genes of Nonstructural Proteins of the Hepatitis C Virus

**DOI:** 10.3390/ijms22158121

**Published:** 2021-07-29

**Authors:** Olga V. Masalova, Ekaterina I. Lesnova, Regina R. Klimova, Alexander V. Ivanov, Alla A. Kushch

**Affiliations:** 1 Gamaleya National Research Center of Epidemiology and Microbiology, Ministry of Health of the Russian Federation, 123098 Moscow, Russia; wolf252006@yandex.ru (E.I.L.); regi.k@mail.ru (R.R.K.); vitallku@mail.ru (A.A.K.); 2 Center for Precision Genome Editing and Genetic Technologies for Biomedicine, Engelhardt Institute of Molecular Biology, Russian Academy of Sciences, 119991 Moscow, Russia; aivanov@yandex.ru

**Keywords:** mesenchymal stem cells (MSC), DNA immunization, hepatitis C virus (HCV), nonstructural HCV proteins, immune response, HCV vaccine

## Abstract

Despite extensive research, there is still no vaccine against the hepatitis C virus (HCV). The aim of this study was to investigate whether MSCs can exhibit adjuvant properties during DNA vaccination against hepatitis C. We used the pcNS3-NS5B plasmid encoding five nonstructural HCV proteins and MSCs derived from mice bone marrow. Five groups of DBA mice were immunized with the plasmid and/or MSCs in a different order. Group 1 was injected with the plasmid twice at intervals of 3 weeks; Group 2 with the plasmid, and after 24 h with MSCs; Group 3 with MSCs followed by the plasmid the next day; Group 4 with only MSCs; and Group 5 with saline. When the MSCs were injected prior to DNA immunization, the cell immune response to HCV proteins assessed by the level of IFN-γ synthesis was markedly increased compared to DNA alone. In contrast, MSCs injected after DNA suppressed the immune response. Apparently, the high level of proinflammatory cytokines detected after DNA injection promotes the conversion of MSCs introduced later into the immunosuppressive MSC2. The low level of cytokines in mice before MSC administration promotes the high immunostimulatory activity of MSC1 in response to a DNA vaccine. Thus, when administered before DNA, MSCs are capable of exhibiting promising adjuvant properties.

## 1. Introduction

Hepatitis C virus (HCV) is a hepatotropic RNA virus that induces chronic liver inflammation, fibrosis, and hepatocellular carcinoma. The HCV burden in public health is estimated at about 71 million people worldwide by the World Health Organization (WHO), with at least 400,000 people that are dying every year from HCV disease [1]. In up to 80% of cases, acute hepatitis C transfers into chronic disease, which may be caused by a very high heterogeneity of the viral genome and the existence of quasispecies, interference of the virus with innate and adaptive immune response pathways, and the formation of “escape” HCV variants that are not recognized by the immune system [2,3]. Despite the breakthrough in the treatment of hepatitis C, it has not yet been possible to determine the exact causes of the frequent chronization of HCV infection, to identify the features of the immune response that determine the elimination of the virus in the acute phase of infection, to fully understand the mechanisms of hepatitis C pathogenesis, or to develop a vaccine. Development of a vaccine against HCV is considered to be one of the main strategies in eliminating the disease and reducing public health burden. However, despite decades of research, there is currently no licensed vaccine against HCV [4]. Effective vaccines can be based on recombinant viral proteins or peptides that contain B- and T-cell epitopes, and DNA plasmids or viral vectors that ensure their expression [4,5]. In case of hepatitis C, it was shown that an effective vaccine should trigger a potent multiepitope and functional Th1 immune response [6]. DNA vaccines induce adaptive immune responses, but naked DNA is weakly immunogenic. Various approaches, including adjuvants, are used to increase the poor immunogenicity of plasmid DNA. Most commonly used are gene adjuvants that encode signaling molecules, such as cytokines, chemokines, immune costimulatory molecules, toll-like receptor (TLR) agonists, and inhibitors of immunosuppressive pathways [7,8]. The spectrum of adjuvants approved by the FDA for use in vaccines is limited. At the same time, the development of new adjuvants with different compositions that could affect the magnitude and quality of the adaptive immune response against specific pathogens is recognized as an important and urgent task [8,9].

One possible approach to enhancement of the response to DNA constructs containing viral genes may be the use of mesenchymal stem cells, also referred to as multipotent mesenchymal stromal cells (MSCs). MSCs have been a focus of recent research, partially because they are an extraordinary model for investigating the biological mechanisms that allow a cellular population to generate diverse cell types and because they are a potential tool in cellular therapies for several clinical applications. MSCs can differentiate into mesenchymal lineages and secrete cytokines and growth factors with paracrine effects that favor the regeneration of damaged tissues [10,11]. A number of studies have established the antifibrotic effect of MSCs in hepatitis [12,13]. According to ClinicalTrials.gov, more than 680 clinical trials using MSCs are registered for cell therapy of many fields, including liver diseases (more than 40 trials) [13]. Advances in cell therapy in recent years are associated with the use of the immunosuppressive properties of MSCs in transplantology, oncology, and some other areas of medicine, although many issues remain unresolved [14]. Depending on the microenvironment, MSCs can exhibit both immunosuppressive and immunostimulatory properties [15]. However, the mechanisms of stimulation of the immune response by MSCs remain unclear and have been studied mostly in vitro in mixed leukocyte reactions [15,16].

We previously showed that modified MSCs carrying the genes of five nonstructural HCV proteins (NS3-NS5) stimulate a cellular immune response in mice that significantly exceeds the immune response to a plasmid encoding the same proteins [17]. The goal of this study was to investigate whether naïve unmodified MSCs can stimulate an immune response during DNA vaccination against hepatitis C, and to determine the conditions under which MSCs exhibit adjuvant properties.

## 2. Results

### 2.1. Characterization of MSCs

As described in detail earlier [17], MSCs obtained were attached to the surface of culture flasks and were polymorphic cells with a fibroblast-like morphology. Phenotyping of MSCs by flow cytometry showed that most of the cells (83–96%) expressed CD73, CD90.1, and CD105 receptors and no expression was detected for hematopoietic cell markers CD45 and CD34. The adipogenic and osteogenic differentiations of MSC were confirmed by detection of neutral fat inclusions or alkaline phosphatase activity, respectively. Thus, in terms of adhesive ability, surface markers, and differentiation potential, the cells meet the minimal criteria for human MSCs of the Mesenchymal and Tissue Stem Cell Committee of the International Society for Cellular Therapy [18]. Numerous studies have shown that the main properties of MSCs isolated from humans are similar to MSCs from other species, including mice [19], rats [20], and dogs [21].

### 2.2. Transfected Huh7.5 Cells and Modified MSCs Express Hepatitis C Virus Genes

**Figure 1:**
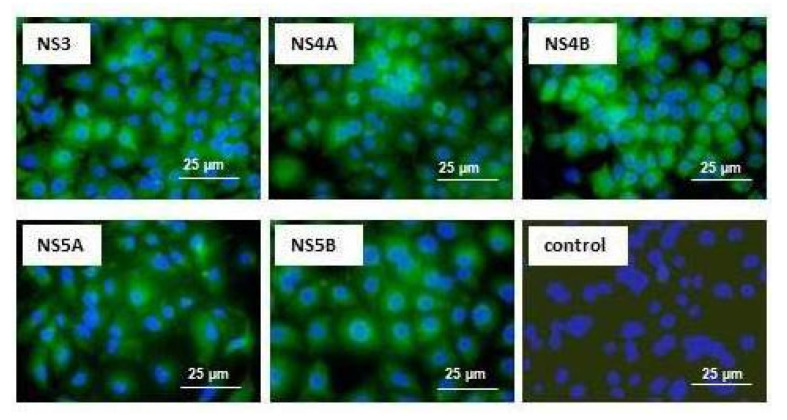
Immunocytochemical staining of hepatitis C virus (HCV) nonstructural proteins in Huh7.5 cells transfected with the pcNS3-NS5B plasmid. Expression of HCV proteins in the transfected Huh7.5 cells was determined by the method of indirect immunofluorescence using monoclonal antibodies (mAbs) against HCV proteins and anti-mouse secondary antibodies conjugated to fluoresceine isothiocianate (FITC; green). The detected HCV proteins are indicated and the mAbs used are listed in Section 4.5. Green staining of HCV proteins is observed in the cytoplasm of cells, merged with nuclear staining with DAPI (blue); no HCV-specific staining was seen in transfected cells stained with mAb to HBsAg (control). Scale bar = 25 µM.

The functional activity of the plasmid was studied with monoclonal antibodies (mAbs). The ability of the plasmid to induce expression of the nonstructural HCV proteins NS3, NS4A, NS4B, NS5A, and NS5B was confirmed by immunocytochemical staining of transfected Huh7.5 cells (Figure 1). Nonstructural HCV proteins reacted with specific mAbs exhibiting antigenic activity and were localized in the cytoplasm; there was no staining in cells stained with non-specific mAbs. It can be noted that all proteins were found predominantly in the perinuclear area, which is in line with numerous reports from other groups.

We also used MSCs transfected with the same plasmid. The expression of nonstructural HCV proteins in MSCs transiently and stably transfected with the pcNS3-NS5B plasmid was shown earlier [17].

### 2.3. Comparative Analysis of Cytokine Production in Cultured and Transfected Cells

**Figure 2:**
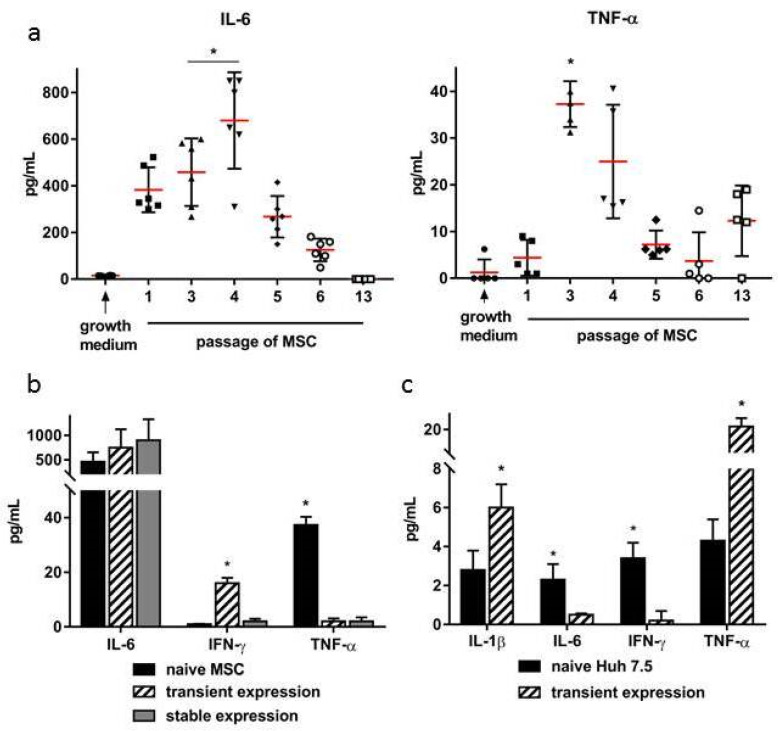
The changes in cytokine levels in a conditional medium during cultivation and transfection of mouse MSCs and human hepatoma cells. (**a**) The concentrations of IL-6 (left) and TNF-α (right) in the conditional medium during the cultivation of MSCs in the dynamics; (**b**) the concentration of cytokines secreted by naïve MSCs (passage 3), MSCs transfected with the pcNS3-NS5B plasmid (72 h), and MSCs stably expressing NS3-NS5B genes (transfection and selection using G-418, day 15); (**c**) comparative level of cytokine production by naïve Huh7.5 cells and cells transfected with the plasmid pcNS3-NS5B (72 h). The concentrations of cytokines are expressed in pg/mL. Values on each diagram are the means ± SD for three independent experiments; * *p* < 0.05 compared to the first passage MSCs (**a**) and naïve non-transfected MSCs (**b**) or Huh7.5 (**c**).

Next, we measured the levels of the cytokines IL-6, IL-10, IL-12, IFN-γ, and TNF-α secreted by MSCs in vitro during serial passages. The concentration of IL-6 in the MSCs conditional medium statistically significantly increased at 3–4 passages, then gradually decreased, and was not detected by the 13th passage. The TNF-α level also reached its maximum values at the same time (Figure 2). IL-10, IL-12, and IFN-γ were not detected in the conditioned medium.

The study of the dynamics of cytokines secreted by modified MSCs at the beginning and at the end of the selection of transfected clones using G-418 (days 3 and 15, respectively) also showed a significant increase in the level of IL-6 (*p* < 0.05) (Figure 2b). In contrast to the naïve MSCs, TNF-α was not detected in the conditioned medium. IFN-γ increased on Day 3 after transfection, but stably transfected MSC cultures did not produce this cytokine.

In Huh7.5 cells transfected with pcNS3-NS5B, the production of cytokines IFN-γ and IL-6 significantly decreased compared to non-transfected cells, while the secretion of the other two—TNF-α and IL-1β—increased (Figure 2c).

Thus, there were significant differences in the production of cytokines IFN-γ, IL-6, and TNF-α between naïve and transfected MSCs on the one hand, and naïve and transfected tumor Huh7.5 cells on the other hand. For immunization of mice, MSCs of 3–4 passages that did not produce IFN-γ were selected, and the secretion of IL-6 and TNF-α was maximal.

### 2.4. Cellular Immune Response to Mitogen

**Figure 3:**
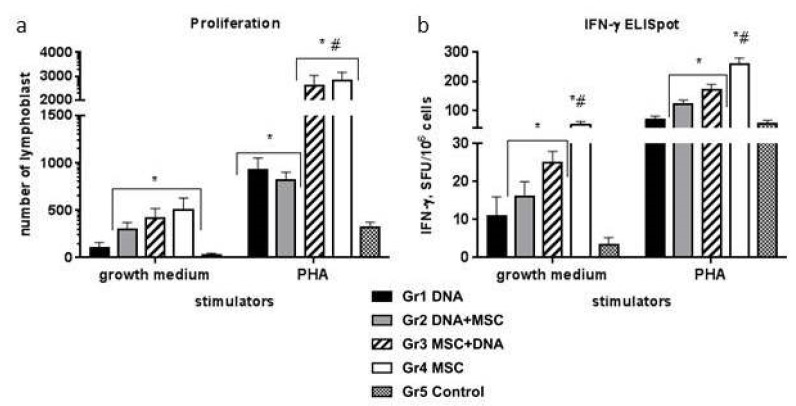
Spontaneous and phytohemagglutinin (PHA)-induced cellular response of lymphocytes from immunized mice in vitro. Each group (5 mice) received the DNA plasmid and/or naïve MSCs during two injections with a three-week interval. To assess the cellular response of the splenocytes in vitro, we used mitogen PHA; the medium alone was used as negative control. (**a**) Results of T-cell proliferation are expressed as the number of lymphoblasts in the cell proliferation reaction; (**b**) the IFN-γ production by splenocytes in response to PHA was assayed as the number of IFN-γ-synthesizing cells by ELISpot as the number of spot forming cells (SFCs) per 10^6^ cells; * *p* < 0.05 compared to the control (Group 5); # *p* < 0.05 compared to all groups.

Mice were immunized with either DNA (pcNS3-NS5B)—Group 1; naïve MSCs—Group 4; or DNA and MSCs in a different order—Groups 2 and 3 (as indicated in Section 4, Materials and Methods, as well as in Figure 3). A week after the second immunization, the cellular immune response to the injected constructs was evaluated. In Groups 2, 3, and 4 of the mice that received MSCs, compared with the control Group 5, spontaneous proliferation of lymphocytes (growth medium) was higher (Figure 3a). In response to phytohemagglutinin (PHA), the number of lymphoblasts increased to a greater extent in Groups 3 and 4 compared to other groups. The appearance of IFN-γ-synthesizing cells had the same tendency: an increase in the number of spots in Groups 2–4 of mice in response to both the medium and PHA (Figure 3b).

Thus, the highest response to PHA was in mice of Groups 3 and 4. Assessment of the proliferative response to mitogens is one of the most universal tests to assess the lymphocyte function; a weak reaction indicates the failure of cellular immunity.

### 2.5. Cellular Immune Response to HCV Proteins

**Figure 4:**
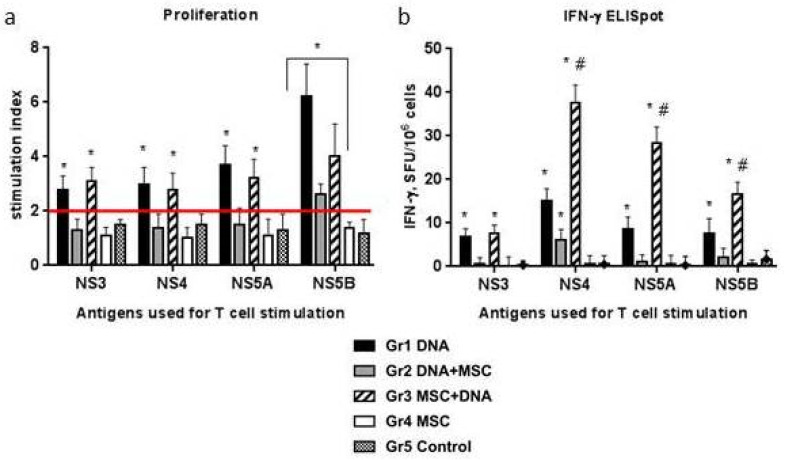
T-cell response of immunized mice to recombinant proteins from the nonstructural region of HCV. Each group (5 mice) received the DNA plasmid and/or naïve MSCs during two injections with a three-week interval. To assess the cellular response of lymphocytes in vitro, we used the recombinant proteins from the nonstructural region of HCV, which were combined into four pools (NS3, NS4, NS5A, and NS5B); the medium alone was used as the negative control. Results of T-cell proliferation are expressed as stimulation indexes (SIs) (**a**); the IFN-γ production by splenocytes in response to HCV proteins was expressed as the difference in the number of spots (spot-forming units, SFUs) per 10^6^ cells between the wells stimulated by the nonstructural HCV antigens and the mean control wells without specific stimulators (growth medium alone) +2 standard deviations (SDs) (**b**). * *p* < 0.05 compared to the control (group 5); # *p* < 0.05 compared to all groups; red line—the threshold level of the reaction.

Specific stimulators—recombinant proteins NS3, NS4B, and NS5A—led to the formation of lymphoblasts only in Groups 1 and 3, and the SI in these groups did not differ (Figure 4a). NS5B protein stimulated lymphocyte proliferation in all groups of plasmid-immunized mice (Groups 1–3), but in Group 2, the SI was significantly lower. No response to HCV proteins was observed in Groups 4 and 5.

In the ELISpot assay, the formation of spots above the control level (the mean number of spots in response to medium + 2 SD) was stimulated by all the proteins used in Groups 1 and 3 and NS4, as well as in Group 2 (Figure 4b). The average number of IFN-γ-synthesizing cells in response to NS4, NS5A, and NS5B proteins was significantly 3–6 times higher in Group 3 compared to Group 1. In Groups 4 and 5, the number of spots was at the background level.

Thus, the cellular immune response to specific HCV stimulators was observed almost only in Groups 1 and 3, while a significant increase in the parameters in Group 3 was only recorded in the ELISpot assay.

### 2.6. The Level of IFN-γ in the Blood Serum of Mice

**Figure 5:**
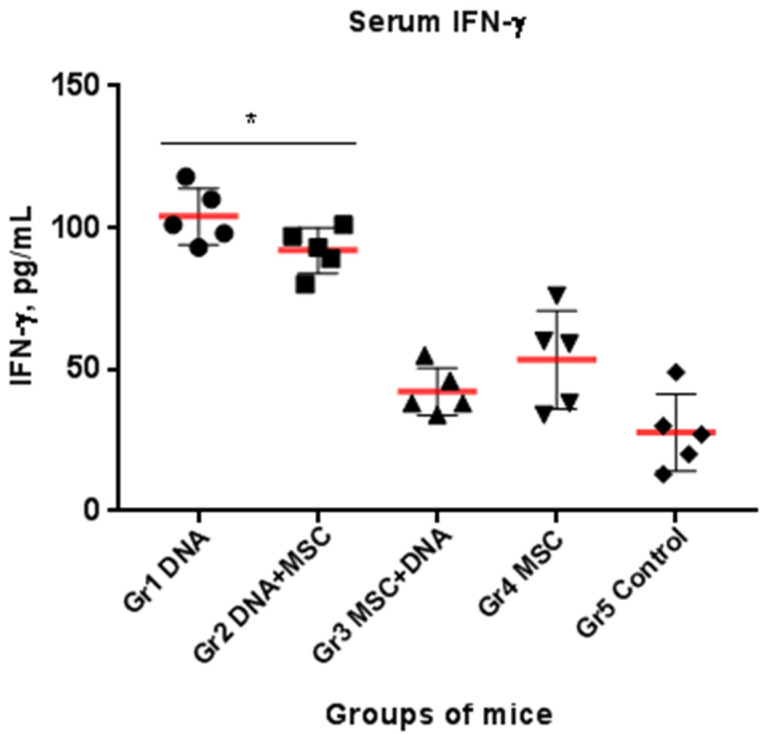
Comparative level of IFN-γ in the blood sera of immunized mice. Each group (5 mice) received the DNA plasmid and/or naïve MSCs during two injections with a three-week interval. After completion of immunizations, the level of IFN-γ in the sera was evaluated by ELISA; * *p* < 0.05 compared to the control (Group 5).

After completion of immunizations, the level of IFN-γ in the sera of the control group mice was 27.8 ± 13.6 pg/mL. In the mice of Groups 1 and 2, it significantly increased more than 3 times (*p* < 0.05); Groups 3 and 4 did not change from Group 5 (Figure 5). Thus, the introduction of HCV DNA to mice leads to an increase in the serum level of IFN-γ, while the introduction of MSCs before the injection of the plasmid reduces it.

## 3. Discussion

During the cultivation of the MSCs obtained from bone marrow, an increase in the production of proinflammatory cytokines IL-6 and TNF-α was observed with a peak at 3–4 passages. The immunological activity of these cytokines can be judged by their antiviral action. Previously, it was shown that the culture fluids from the MSCs on the fourth passage, containing the maximum concentrations of IL-6 and TNF-α, showed the maximum effect of suppressing the infectious activity of the herpes simplex virus (HSV-1) in vitro [22]. Therefore, to study the immunostimulatory activity of MSCs in vivo, we injected mice with MSCs collected at 3–4 passages of cultivation, when the functional activity and proinflammatory properties of the MSCs were high. A comparison of the production of proinflammatory cytokines by MSCs and Huh7.5 hepatocarcinoma cells showed significant differences: the TNF-α and IL-6 concentrations were 10 and 200 times higher in the MSCs, while IFN-γ was produced only by Huh7.5 cells. The reaction to pcNS3-NS5B DNA transfection also appeared to be different: the production of IL-6 and IFN-γ increased in the MSCs, decreased in Huh7.5, and the concentration of TNF-α significantly increased only in Huh7.5 cells. These data can be explained by the different nature of the compared cells: normal mouse bone marrow cells and human tumor cells. Huh7.5 cells have mutations in some genes including the *RIG-1* gene that encodes a cytoplasmic sensor for viral RNA, and can initiate anti-viral and inflammatory cell responses [23]. It is possible that the reduced expression of IL-6 and IFN-γ in Huh7.5 cells transfected with pcNS3-NS5B is due to the low activity of RIG-1, an interferon-inducible cellular DExD/H box RNA helicase.

**Figure 6:**
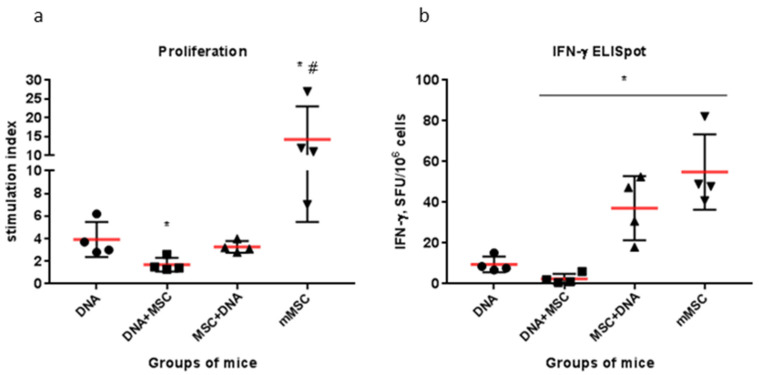
Comparative efficacy of the overall mean cellular responses to HCV in different variants of DNA immunization using MSCs. Mice were immunized with pcNS3-NS5B plasmid alone or in various combinations with MSCs. To assess the overall mean cellular response of lymphocytes in vitro to recombinant HCV proteins, we averaged the data obtained for the recombinant proteins from the nonstructural region of HCV, which were combined into four pools (NS3, NS4, NS5A, and NS5B). The overall mean cellular response to HCV proteins in the lymphocyte proliferation reaction (**a**), and in the number of IFN-γ-synthesizing cells by ELISpot (**b**). DNA—naked pcNS3-NS5B plasmid; MSC—naïve MSC; mMSC—genetically modified MSC expressed nonstructural HCV proteins [17]. * *p* < 0.05 compared to the naked DNA group; # *p* < 0.05 compared to all groups.

The data on the different effects of MSCs on DNA immunization are of the greatest interest. We assume that it is due to the different order of administration of MSCs—before or after the plasmid immunogen. It was shown that the intramuscular introduction of naked plasmid DNA leads to effective expression of the transgene in the skeletal muscle [24]. In the model system, it was found that the plasmid immunogen delivered by the intramuscular injection leads to early (after 2 h) expression of the immunogen [25]. Then, the transgene release derived the peptides/proteins via exosomes or apoptotic bodies. This material is endocytosed by immature dendritic cells, which subsequently present antigens preferentially via the major histocompatibility class (MHCII) to CD4+ T-cells in draining lymph nodes [8]. Several types of antigen-presenting cells (APCs) recognize external pathogens and subsequently initiate defensive immune mechanisms. In our work, the intramuscular administration of a plasmid containing and expressing five HCV genes elicited an immune response to HCV, inducing T-cell proliferation and IFN-γ synthesis. MSCs administered intravenously 24 h after recombinant DNA suppressed both of these parameters by an average of 2.5 and 5 times, respectively (Figure 6). We also showed that the expression of HCV proteins during transfection of mouse cells with plasmid was accompanied by an increase in the level of proinflammatory cytokines, as well as an increase in IFN-γ in the sera of immunized mice in the case of injection of DNA alone or DNA before MSCs. The immunosuppressive effect of MSCs in this case can be compared with that observed when using MSCs for the treatment of patients with COVID-19 [26]. During the inflammatory response, many proinflammatory cytokines are secreted, including TNFα, IL-1β, IL-2, IL-6, IL-7, IL-12, IL-18, IL-33, IFN-α, and IFN-γ. An excessive increase in reactivity leads to a cytokine storm—the leading mortality factor due to SARS-CoV-2 infection [27,28,29]. A large number of experimental and clinical studies demonstrated that injection of MSCs or their secretome significantly reduces inflammation and the expression of chemokines and proinflammatory cytokines and enhances regeneration and functional recovery [27,30]. MSCs also have been used in clinical trials to treat patients infected with influenza virus A (H7N9), which had symptoms similar to those in patients infected with SARS-CoV-2 [31], and in experiments with influenza viruses H5N1 and H9N2 [32,33]. Stem cells are also being tested to treat other viral infections, such as hepatitis B, HIV-1, and Coxsackie B3 [34,35,36].

The mechanisms of the immunosuppressive properties of MSCs have been analyzed in many studies. MSCs are reported to inhibit the immune response from innate immune cells, including monocytes and macrophages [37], dendritic cells (DCs) [38], natural killer cells (NK cells) [39], and that from adaptive immune cells, including T-cells [40], B-cells [41] and other immune cells [42]. In vitro, MSCs can stop a variety of immune cell functions, namely, cytokine secretion and the cytotoxicity of T- and NK cells, B-cell maturation and antibody secretion, DC maturation and activation, and antigen presentation. Some authors believe that the immunosuppressive properties of MSCs are mediated by cell surface receptors [43]. In contrast, others reported a reduction in the immunosuppressive activity of MSCs that promoted the T-cell and neutrophil survival, activation, and response upon TLR ligation [44,45]. MSCs affect the functions of most immune effector cells via direct contact with immune cells or indirectly via local microenvironmental factors that modulate APCs and other accessory cells. Previous studies have confirmed that the immunomodulatory effects of MSCs are mainly communicated via MSC-secreted cytokines; however, apoptotic and metabolically inactivated MSCs have more recently been shown to possess immunomodulatory potential, in which regulatory T-cells and monocytes play a key role [46]. It should be noted that in vivo studies have shown many discrepancies regarding the mechanisms of the immunomodulatory properties of MSCs [47].

Much less is known about the proinflammatory properties of naïve and engineered MSCs, which are associated with the attraction and stimulation of granulocytes, macrophages, NK cells, and proinflammatory cytokine induction in vitro [9,16,48,49] and in vivo [17,50,51,52,53]. One of the explanations for the multidirectional actions of MSCs is the polarization of MSCs. The concept of MSC polarization into proinflammatory and anti-inflammatory cells provides an attractive model to explain and investigate the apparently contradictory roles of MSCs in inflammation [54]. Two different immune phenotypes have been described for MSCs, depending on which toll-like receptor (TLR) is activated. MSC1 is endowed with a proinflammatory phenotype following TLR4 activation with LPS. On the other hand, anti-inflammatory MSC2 is induced by the activation of TLR3 with Poly(I:C) [55,56,57]. MSC1 shows an increased synthesis and secretion of proinflammatory cytokines and chemokines, such as IL-6 and IL-8, whereas MSC2 has increased production of immunosuppressive mediators, such as IP-10 and CCL5. MSCs have high immunoplasticity, and the phenotype conversion can be caused by exogenous stimuli, such as proinflammatory cytokines or TLR agonists, as well as the duration of treatment [15,57,58]. The data on the effect of exogenous proinflammatory cytokines on MSCs are contradictory. Several authors have noted an increase in the antigen-presenting properties of MSCs as a result of IFN-γ pretreatment [59]. Other studies have shown that the “priming” of MSCs in vitro with IFN-γ, TNF-α, or IL-1β leads to the formation of the immunosuppressive phenotype MSC2 [15,58,60]. We administered MSCs of 3–4 passages that did not produce IFN-γ, and the secretion of IL-6 was maximal, to healthy mice. It was shown that the accumulation of IL-6 leads to the activation of the MSC1 population and promotes the formation of Th17 cells that activate the immune response [15]. Apparently, an increase in the level of pro-inflammatory cytokines as a result of HCV protein expression in Group 2 mice in response to plasmid administration contributed to the transformation of MSCs into the MSC2 phenotype with suppressive activity on the adaptive immune response to HCV.

Another explanation of the phenomenon described by us may be related to the effect of MSCs on one of the populations of suppressor cells—myeloid suppressor cells (MDSCs). They represent a heterogeneous population of immature myeloid cells with a powerful suppressor potential. The role of MDSCs in viral infections has not been adequately studied [61]. In patients infected with HCV, an increase in the MDSC population is observed; these cells inhibit the proliferation of CD4+ and CD8+ lymphocytes, NK cells, and IFN-γ production [62,63]. Previously, we found that MSCs caused a 2-fold reduction in the number of MDSCs in immunized mice compared to groups of control mice and those immunized with the DNA vaccine [17]. Thus, one of the mechanisms of stimulation of the innate and adaptive immune response by MSCs in our experiments may be the suppression of MDSCs. Interestingly, when modeling cancer in mice, a dependence of the immunomodulatory “phenotype” of MSCs on the injection site was found. The simultaneous injection of MSCs with tumor cells led to immunosuppression, and distal injection led to immunostimulation; the immune response was shown to correlate with a decrease in the proportion of MDSCs and regulatory T-cells [64].

A comparison of the data in Figure 3 and Figure 4 shows that the T-cell response to the mitogen in Group 3 (MSCs + DNA) is higher in both proliferation and IFN-γ production than the specific response to HCV proteins. At the same time, the differences in the proliferative response were significantly greater than in the synthesis of IFN-γ. Thus, not all proliferating cells induced by PHA are able to synthesize IFN-γ. The specific response to HCV proteins, on the contrary, manifests itself in an increase in the production of IFN-γ with an unchanged level of T-cell proliferation. It was of interest to compare the specific T-cell response in groups immunized with naïve MSCs with similar response rates to genetically modified MSCs (mMSC) expressing the same HCV proteins obtained earlier [17]. The overall mean responses to all HCV proteins used in different variants of DNA immunization were compared (Figure 6). It turned out that, in the mouse group immunized with MSCs before DNA, the production of IFN-γ was as high as in the mMSC immunization (Figure 6b). At the same time, the proliferative response in the mMSC group was significantly higher (Figure 6a). The main method that is recommended for assessing the response of T-cells to new HCV vaccines is the quantification of IFN-γ production by the ELISpot method, which shows the activity of the antiviral response [5,65,66]. Therefore, the data obtained using naïve MSCs to improve the effectiveness of DNA immunization are very important.

## 4. Materials and Methods

### 4.1. Mice

Mice of the DBA/2J (H-2d) line (females, 6–8 weeks old) were obtained from the laboratory of the animal breeder Stolbovaya, FMBA, Moscow Region. All ex vivo and in vivo animal experiments were carried out in accordance with the order 199n of the Ministry of Health of the Russian Federation and with the “Regulations on the ethical attitudes to laboratory animals of N.F. Gamaleya NRCEM (Moscow, Russia)”.

### 4.2. Isolation and Characterization of Primary MSCs

The primary culture of MSCs was obtained by isolation from the red bone marrow of the femur bones of DBA mice as described earlier [17]. Briefly, mouse primary MSCs were obtained from bone aspirates of DBA mice. The cell suspension was homogenized and centrifuged at 2000× *g* for 10 min. Cell pellets were resuspended in high glucose Dulbecco’s modified Eagle medium (DMEM) containing 10% fetal calf serum (FCS) (Invitrogen, Waltham, MA, USA), 10 µg/mL insulin, 5.5 µg/mL transferrin, 6.7 ng/mL sodium selenite, 10 ng/mL basic fibroblast growth factor, 2 mM L-glutamine, and 50 µg/mL gentamicin. The cells were seeded in culture flasks (Costar, New York, NY, USA) at a concentration of 2 × 10^6^ cells/mL. The next day, as well as every subsequent 3–4 days, the culture medium was replaced. The resulting adhesive cell population was reseeded using a 0.25% trypsin solution. MSCs were cultured at 37 °C in a 5% CO_2_ atmosphere. Unless otherwise specified, culture media and other reagents were purchased from PanEco, Russia (Moscow, Russia).

The cells were characterized by morphological properties, adhesive ability, expression of surface markers, and the potential for adipogenic and osteogenic differentiation, as described above [17].

### 4.3. Cell Line

The human hepatoma Huh7.5 cell line [67] was cultured in Dulbecco’s modified minimal essential medium (Paneco, Moscow, Russia) supplemented with 10% fetal calf serum (Gibco, Waltham, MA, USA), 2 mM glutamine, and 50 µg/mL gentamycin at 37 °C in a humid atmosphere with 5% CO_2_.

### 4.4. Plasmid and Transfection

We used the pcNS3-NS5B plasmid construct encoding five nonstructural HCV proteins (NS3, NS4A, NS4B, NS5A, and NS5B) of genotype 1b that was constructed using a commercially available pcDNA-3.1(+) vector (Invitrogen, USA) [68]. The plasmid was purified from *E. coli* strain JM109 using a commercial QIAGEN Plasmid Purification Maxi Kit (QIAGEN, Hinden, Germany) according to the manufacturer’s instructions. To confirm the plasmid functionality, Huh7.5 cells were transfected using TurboFect Transfection Reagent (Thermo Fisher Scientific, Rockford, IL, USA), as described above [69]. MSCs of mice were transfected with the same plasmid using Xfect Transfection Reagent (Clontech Laboratories, Takara, San Jose, CA, USA), and a stably transfected MSC (mMSC) line was obtained using the G-418 selective antibiotic, as described earlier [17].

Cytokine secretion was measured by quantifying the cytokine levels in the conditioned medium, as described below.

### 4.5. Immunocytochemical Detection of HCV Proteins

Expression of HCV proteins in the transfected Huh7.5 cells was determined by the methods of indirect immunofluorescence, using original monoclonal antibodies (mAbs) against HCV proteins (2H4 to NS3, 3F12 to NS4A, 6B11 to NS4B, and 3F4 to NS5A) [70], and commercially available mAbs to the NS5B protein (sc-58146, Santa Cruz Biotechnology, Dallas, TX, USA), as previously described [69]. As secondary antibodies, fluorescein isothiocyanate-labeled (FITC) anti-mouse IgGs (Dako, Denmark) were used. Nuclei were stained with 4′-6-diamino-2-phenylindole dye (DAPI). Staining was visualized using an Axio Scope A1 Carl Zeiss (Jena, Germany) fluorescent microscope at excitation/emission wavelengths of 520/560 nm and 360/460 nm (DAPI) at 400× magnification.

### 4.6. Immunization of Animals

Five groups of mice were immunized with pcNS3-NS5B plasmid and/or MSCs of 3–4 passages in a different order (5 animals per group). The mice from Group 1 (DNA) were injected with plasmid; from Group 2 (DNA + MSC) with plasmid, and after 24 h with MSCs; from Group 3 (MSC + DNA) first with MSCs and the next day with plasmid; from Group 4 (MSC) only with MSCs; and from Group 5 (Control) with saline solution. MSCs (5 × 10^5^ cells) were injected into the tail vein, and plasmid (100 µg) was administered intramuscularly into the quadriceps femoris muscle. Two immunizations with an interval of 3 weeks were conducted.

### 4.7. The Recombinant HCV Proteins

The recombinant HCV proteins were used as antigens to stimulate T-cell responses in vitro. The proteins were combined into four pools: NS3 (helicase domain with a sequence of 1230–1658 aa, immunodominant region 1356–1459 aa, genotype 1b); NS4 (1677–1754 aa and mosaic protein containing regions 1691–1710, 1712–1733, 1921–1940 aa from genotypes 1, 2, 3, and 5); NS5A (the full-length protein 1973–2419 aa and fragments 2061–2302 aa, 2212–2313 aa, genotypes 1b and 1a); and the NS5B protein lacking C-terminal hydrophobic 21 amino acid residues (2420–2990 aa, genotype 1b). The recombinant proteins were expressed in *E. coli* and purified by chromatography on Ni-NTA-agarose or on glutathione sepharose, as described previously [68,71,72,73].

### 4.8. T-Cell Proliferation and ELISpot Assays

A week after the second immunization, the cellular immune response to the injected constructs was evaluated. T-cell proliferation in vitro was assessed by activation of the DNA synthesis as described previously [74], with minor modifications. The spleens of 5 mice of each group were pooled, a suspension of splenocytes was seeded in U-bottomed 96-well microculture plates at a density of 5 × 10^5^ cells/well, and specific stimulants (pools of the recombinant HCV NS3, NS4, NS5A, and NS5B proteins at a final concentration of 1 µg/mL) were added. As negative controls, we used the medium alone (spontaneous proliferation); mitogen phytohemagglutinin (PHA, 5 µg/mL, Sigma, St. Louis, MO, USA) was used as an unspecific positive control. All samples were set in at least four replicates. The cells were cultured in a RPMI-1640 medium containing 20% FCS (Invitrogen, Waltham, MA, USA), 4.5 mg/mL glucose, 2 mM glutamine, 0.2 u/mL insulin, and 50 µg/mL gentamicin at 37 °C in a 5% CO_2_ atmosphere. Splenocyte proliferation was assessed using the blast transformation test after 2 days for PHA and 6 days for HCV antigens. The results were presented as the stimulation index (SI) calculated as the ratio of the average lymphoblast numbers observed in the presence and in the absence of specific stimulators. A positive result was registered at SI > 2.

Quantification of cells secreting IFN-γ was carried out with the ELISPOT mouse IFN-γ Kit (BD Biosciences, San Jose, CA, USA) in accordance with the manufacturer’s instructions. Stained spots were visualized using an MBS-10 stereo microscope (LOMZ, Russia). The results were expressed as the difference in the number of spots (spot-forming units, SFUs) per 10^6^ cells between the wells stimulated by the nonstructural HCV antigens and the mean control wells without specific stimulators (growth medium alone) +2 standard deviations (SDs).

### 4.9. Detection of Cytokines in Cell Culture Media and Mouse Sera with Sandwich ELISA

Measurement of the mouse cytokine levels (IFN-γ, TNF-α, IL-6, IL-10, IL-12) was performed with ELISA in conditioned medium from MSCs during 1–13 passages and also in a medium from a selection of the MSCs transfected with pcNS3-NS5B. IFN-γ quantification was also conducted in mice sera after second immunization. We used the Mouse IL-6 ELISA development kit (HRP), Mouse IFN-γ ELISA development kit (HRP), Mouse TNF-α ELISA development kit (HRP) (Mabtech, Stockholm, Sweden), Mouse IL-10 DuoSet ELISA, and Mouse IL-12 p70 Duoset ELISA (R&D Systems, Minneapolis, MN, USA). The detection sensitivity for IL-6 was 10 pg/mL, for IFN-γ and TNF-α 2 pg/mL, and for IL-10 and IL-12 30 pg/mL.

Human cytokine secretion by Huh7.5 cells was assayed by ELISA, using Vektor-Best kits (Russia) for IL-1β, IL-6, IFN-γ, and TNF-α. The sensitivities of the cytokine assays were 0.5 pg/mL for IL-6, 1 pg/mL for IL-1β and TNF-α, and 2 pg/mL for IFN-γ. The concentrations of cytokines were determined from the calibration curves of the standard samples.

### 4.10. Statistical Analysis

Statistical analysis was performed using Statistica 8 (StatSoft Inc., Tulsa, OK, USA) and GraphPad Prism 7 (GraphPad5, SanDiego, USA) software. The data are presented as the means ± SD of three independent experiments and analyzed by two-tailed Student’s *t*-test or one-way analysis of variance (ANOVA), followed by Tukey tests for multiple comparisons when appropriate (*p* < 0.05 was considered as statistically significant).

## 5. Conclusions

The development of a DNA vaccine against hepatitis C involves many different research directions, including the search for the optimal adjuvant. Our study showed for the first time that bone marrow naïve MSCs can exhibit both stimulating and inhibitory effects on the same DNA immunogens. When administered before DNA in preventive DNA immunization, the number of lymphocytes synthesizing IFN-γ in response to HCV significantly increased. We consider our results as a basis for further preclinical studies of the protective effect of naïve MSCs as well as genetically modified MSCs in the future. Further studies are required for a detailed study of the mechanisms of the immunostimulatory action of naïve MSCs. At the same time, the large resources of MSCs, their availability in large quantities, and their proven safety indicate that MSCs can serve as the basis for the development of an effective adjuvant for a vaccine against hepatitis C and other viral infections, and hopefully, the problems associated with their the immunocompatibility, stability, and heterogeneity can be solved in the future.
